# Dissociable Effects of Theta-Burst Repeated Transcranial Magnetic Stimulation to the Inferior Frontal Gyrus on Inhibitory Control in Nicotine Addiction

**DOI:** 10.3389/fpsyt.2020.00260

**Published:** 2020-04-15

**Authors:** Roger D. Newman-Norlund, Makayla Gibson, Patrick A. McConnell, Brett Froeliger

**Affiliations:** ^1^ Department of Psychology, University of South Carolina, Columbia, SC, United States; ^2^ Department of Neuroscience, Medical University of South Carolina, Charleston, SC, United States; ^3^ Hollings Cancer Center, Medical University of South Carolina, Charleston, SC, United States

**Keywords:** fMRI, TBS, TMS, tobacco, inhibition, executive, brain, cognition

## Abstract

Nicotine addiction, like other substance use disorders (SUD’s), is associated with deficits in prefrontal mediated inhibitory control. The strength of inhibitory control task-based functional connectivity (tbFC) between the right inferior frontal gyrus (r.IFG) and thalamus (corticothalamic circuit) mediates the association between successful inhibition and smoking relapse vulnerability. However, the potential efficacy of theta burst stimulation (TBS) to the r.IFG, a treatment known to alter clinical symptoms among neuropsychiatric patients, has not been reported in a SUD population. This study utilized fMRI guided neuronavigation to examine the effects of TBS on inhibitory control among nicotine dependent individuals. Participants (*N*=12) were scanned while performing an inhibitory control task known to elicit inhibition-related activity in the r.IFG. Using a randomized, counterbalanced cross-over design, participants then received TBS over two visits: excitatory (iTBS) on one visit and inhibitory (cTBS) TBS on the other visit. The effects of each TBS condition on subsequent inhibitory control task performance were examined. A significant condition x time interaction was identified on trials requiring inhibitory control (F (1,10) = 7.27, *p* = .022, *D* = 1.63). iTBS improved inhibitory control, whereas cTBS impaired inhibitory control. Brain stimulation did not influence performance in control conditions including novelty detection and response execution. This is the first study to demonstrate that non-invasive neural stimulation using iTBS to the r.IFG enhances baseline inhibitory control among individuals with a SUD. Further research is needed to directly examine the potential parametric effects of TBS on corticothalamic tbFC in individuals with a SUD.

## Introduction

Substance use disorders (SUDs) are characterized by significant disruptions to multiple forms of executive function (1) and the extant literature implicates dysregulated inhibitory control, one specific form of executive function, as a transdiagnostic indicator of relapse vulnerability across substances of abuse (2). In the context of tobacco use disorder (nicotine addiction), smokers, as compared to non-smokers, exhibit significantly worse performance on tasks that probe inhibitory control (IC) (3–5); and among smokers, smoking abstinence as compared to satiety, further disrupts inhibitory control task performance (6, 7). Furthermore, baseline inhibitory control task performance is significantly associated with smoking outcomes following a quit attempt (8, 9) and the capacity to resist ad lib smoking in a laboratory setting (9, 10). Despite compelling evidence of IC deficits contributing to the maintenance of nicotine addiction, there is little support for the therapeutic value of existing first line FDA approved smoking cessation medications for treating inhibitory control mechanisms (11, 12). Therefore, there is a need for mechanistic research to identify new strategies for treating IC pathophysiology in tobacco use disorder.

The extant literature converges on a role of the right inferior frontal gyrus (r.IFG) as a key prefrontal region involved in the initiation of a “stop” command (13, 14). Individuals with a substance use disorder (2), including tobacco use disorder (15–17), exhibit greater fMRI BOLD response in the r.IFG during task probes of attention (18) and inhibitory control (6) that may represent a compensatory mechanism to meet task demands. Given strong evidence for the involvement of r.IFG in inhibitory control and smoking related dysregulation in r.IFG mediated inhibition, examining whether neural stimulation to the r.IFG can modulate inhibitory control in smokers represents an important avenue for examination.

Thetaburst stimulation, a form of patterned transcranial magnetic stimulation (tb-pTMS) can be used to modulate neuronal function within a focused area, as well as functionally connected brain regions. TBS has a good safety profile (19, 20) and is administered in two forms: a) Intermittent TBS (iTBS) (21) which enhances spontaneous neuronal firing (22) and induces long-term potentiation (LTP)—putatively strengthening neural activity (23); and b) Continuous TBS (*c*TBS) (24) which induces long-term depression—putatively dampening neural activity. The effects of LTD-like *c*TBS to r.IFG have been reported in two studies with healthy controls, demonstrating that *c*TBS disrupts inhibitory control task performance (25, 26), but not performance in other, task-related domains (general attention, generic responding) (25). However, to the best of our knowledge, there is no published work, either in a healthy or in a clinical sample, on the effects of iTBS—the pattern with potential therapeutic value—to r.IFG on inhibitory control.

Therefore, given the lack of known medications for treating inhibitory control deficits among smokers and the unknown potential of iTBS for modulating inhibitory control among individuals with an SUD, the goal of the current study was to examine the effects of TBS on inhibitory control task performance in smokers. To this end, r.IFG regions involved in inhibitory control were identified for each individual (N = 12) using fMRI data acquired during performance of a validated IC test. Using a crossover design, we examined the effect of both excitatory (iTBS) and inhibitory (cTBS) TBS to the r.IFG on inhibitory control task performance in each individual. We hypothesized that the excitatory stimulation would improve, and that the inhibitory would disrupt, IC task performance. The effects of TBS on novelty detection and generic motor responding were also assessed.

## Materials and Methods

### Participants

Twelve healthy adult (age: *M* = 31.42 years ± 7.39, three females, education: *M* = 13.00 years ± 1.35) nicotine dependent (FTND: *M* = 5.42 ± 2.19) smokers, smoking on average 16.42 ± 4.52 cigarettes per day for 13.83 ± 7.57 years completed the study (see **Table 1**). Inclusion criteria were being in good health, right-handed, aged 18-55 years, and smoking ≥ 10 cigarettes/day. Exclusion criteria were having significant health problems, contraindications for MRI, use of psychoactive medications, smokeless tobacco or nicotine replacement therapy (NRT), current drug or alcohol abuse, afternoon expired carbon monoxide (CO) level < 10 ppm (Vitalograph Inc., Lenexa KS), breath alcohol level (Alert breathalyzer; Columbia Laboratory Supplies), or urine pregnancy test. The study was approved by the institutional review boads of the Medical University of South Carolina and the Univeristy of South Carolina. Participants were recruited *via* local media outlets in Columbia, SC, expressed no interest in quitting smoking, gave full written informed consent and received financial compensation for study participation.

**Table 1:** Subject demographics/baseline assessments.

Sample N (Female)	12 (3)
Mean age	31.42 (7.39)
Years of education	13.00 (1.35)
Smoking related variables	
Nicotine dependence (FTND)	5.42 (2.19)
Years smoking	13.83 (7.57)
Average daily cigarettes	16.42 (4.52)
Carbon monoxide (CO): Visit 1	19.50 (8.73)
Minutes since last cigarette: Visit 1	23.75 (16.11)
Carbon monoxide (CO) Visit 2	23.33 (9.20)
Minutes since last cigarette: Visit 2	15.83 (15.49)
Positive urine cannabis screen	6

Standard deviation reported in parentheses next to mean where applicable.

### General Study Procedures

**Figure 1:**
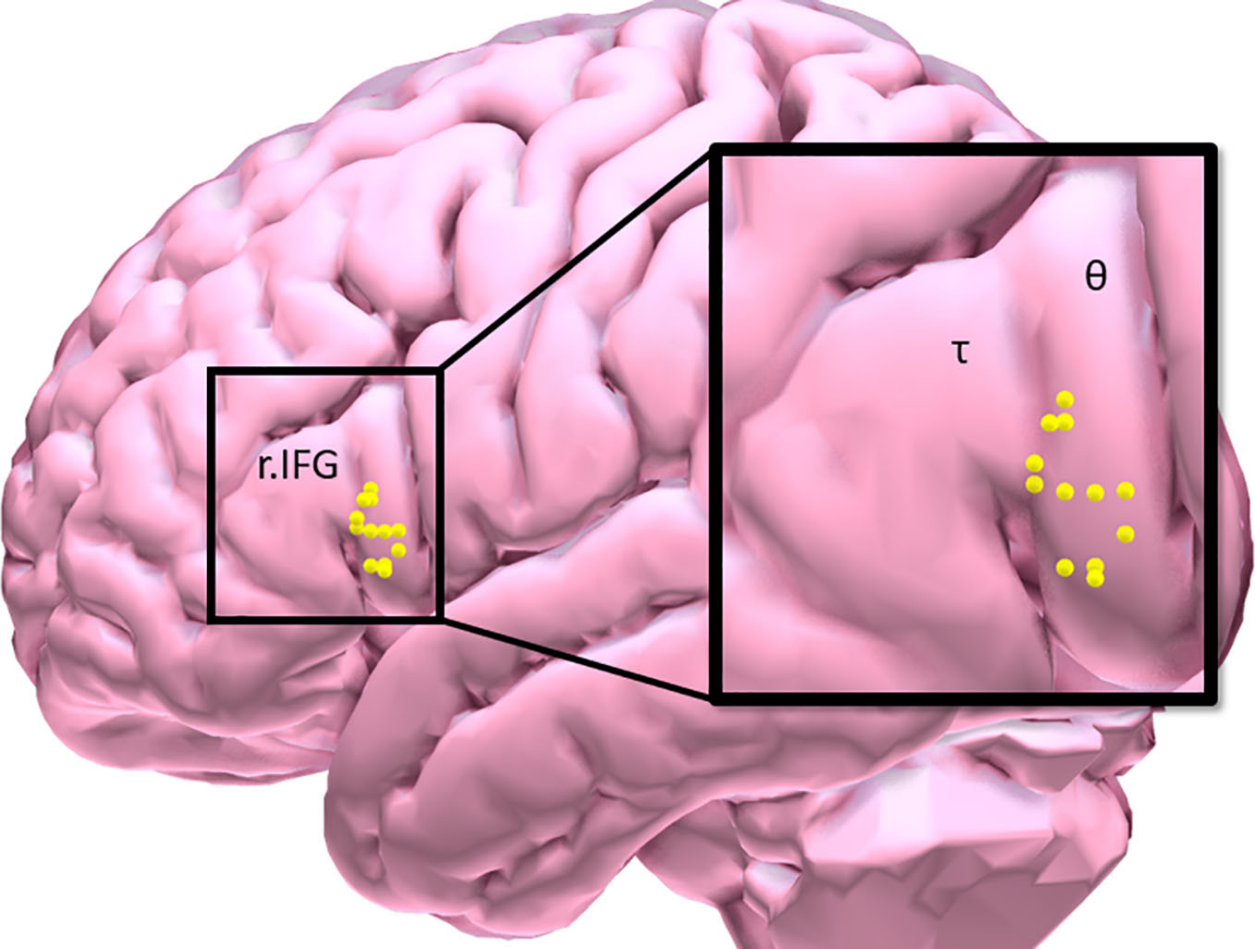
Participant distribution of maximal inhibitory control task-related BOLD response in the right inferior frontal gyrus [τ = triangularis (*n* = 3); θ = opercularis (*n* = 9)].

Eligible participants underwent a brief training visit to learn and practice the inhibitory control task, complete a smoking-history questionnaire and the Fagerstrom Test of Nicotine Dependence (FTND). FTND scores ranged between 1 (low) and 9 (high) (27); and a urine cannabis screen was administered to assess recent use. Participants were tested while in a smoking satiated state during each experimental visit (i.e. < 30 min since smoking a cigarette of their preferred brand). Participants first underwent an experimental fMRI visit during which time they performed the IC task in order to measure baseline inhibitory control task performance and identify an individual’s task-related peak activation cluster in the r.IFG [BA 44 and 45: see **Figure 1**, **Table 2**]. Using a randomized, counterbalanced cross-over design, participants then returned for two separate experimental TMS visits (separated by 2–30 days) and received iTBS on one visit and cTBS on the other visit. During each of the two experimental TMS visits, all participants performed the inhibitory control task at the beginning of the session on a computer outside of the scanner. Following a 15-min break, the appropriate TBS protocol was performed and, after a 15-min delay, participants again performed the inhibitory control task on a computer outside of the scanner. The effects of TBS stimulation on inhibitory control task performance were assessed by examining changes in behavioral task performance from 15 min pre- to- 15 min post-TBS.

**Table 2:** MNI coordinates of the peak activation in the r.IFG for each participant.

Participant	x	y	z	SubRegion
N1	52	10	5	opercularis
N2	56	20	13	triangularis
N3	53	23	3	triangularis
N4	51	12	8	opercularis
N5	55	13	8	opercularis
N6	55	12	14	opercularis
N7	55	19	13	triangularis
N8	57	15	10	opercularis
N9	57	6	8	opercularis
N10	53	14	8	opercularis
N11	53	12	3	opercularis
N12	55	13	3	opercularis

### Inhibitory Control (IC) Task

The validated event-related IC task (9, 28) included randomly presented colored circles—frequent gray (“Go”; 75.4%; n=388), rare yellow (“RareGo”; 12.3%; n=65), and rare blue (“NoGo” 12.3%; n=65). Participants were instructed to use their right index finger to press a button as quickly as possible for “Go” and contextually novel “RareGo” trials and to refrain from pressing in response to contextually novel “NoGo” trials. Behavioral data were processed and analyzed consistent with our prior work with this task (9). Prior to analysis, NoGo performance was corrected by scoring NoGo trials with a null response as incorrect when the participant did not respond to the “Go” trial immediately preceding it. The rare go trials are a particularly important component of this task, as they provide a novelty detection control condition to compare with the novel inhibitory control trials.

### Image Acquisition and Modeling

Imaging was performed on a 3T Siemens Prisma scanner: a high-resolution 3D MPRAGE anatomical sequence was acquired (matrix = 256, flip angle = 9°, 166 slices, 1mm isotropic voxels; whole brain BOLD contrast sensitive images were acquired using a multi-band (6) EPI sequence (60 slices, TR=800 ms, TE=30 ms, FOV=216, 2.4 mm isotropic voxels).

#### fMRI IC Task Data Preprocessing

Similar to our prior analytic strategy using this task (9), fMRI data were preprocessed using SPM12 to remove noise and artifacts, motion corrected (29), temporally realigned using B-spline interpolation and smoothed with an 8 mm FWHM Gaussian filter. Functional images for each participant were processed in their native space.

#### IC Task Modeling

Preprocessed data were entered into a first-level, whole-brain analysis using the General Linear Model to examine BOLD response to each of the five trials of interest: NoGo_correct_ (successful inhibition), NoGo_incorrect_ (error of commission), RareGo_correct_ (novel-target detection), RareGo_incorrect_ (novel-target error of ommission), and Go_incorrect_ (error of omission). Each event was modeled as a delta regressor (onset dur. = 0) and convolved with a canonical hemodynamic response function. Motion was removed through rigid body rotation and translation and parameters included as covariates. A high-pass filter (128 s; .008 Hz) was applied to remove slow signal drift. To idenitfy successful IC-BOLD response, controlling for novelty detection, a NOGO_correct_–RareGo_correct_ contrast image (IC-contrast) was generated (9) and fed forward to ROI identification for neuronavigation. IC task BOLD response was collected only during the baseline visit and used to inform the subsquent neuronavigation protocol (see **Figure 1**, **Table 2**).

### Neuronavigation Protocol

Following the baseline scan and ROI identification procedure performed by BF (**Figure 1**, **Table 2**), neuronavigation-based TBS was administered using the Rogue Research Inc. ^©^ Brainsight system. First, co-registered anatomical and functional ROI data were entered into a participant’s workflow profile. Skin and full-brain curvilinear reconstructions were created and external landmarks (bridge of nose, l.ear, r.ear) were created. Finally, the r.IFG was set as the spatial target, and the target coil trajectory was set. The same setup parameters were used across each of the two TBS visits.

### Theta Burst Stimulation Protocols

Participants were randomized to receive TBS to the r.IFG on two separate experimental visits: iTBS on one visit; cTBS on one visit—counterbalanced across participants.

#### Determining Resting Motor Threshold

Standard procedures were used to determine the participants resting motor threshold (RMT) using parameter estimation by sequential testing (PEST) procedures (30).

#### Intermittent Theta Burst Stimulation (iTBS) to the r.IFG

The total duration of the iTBS protocol (21) was 190 s. Participants received stimulation over the r.IFG (a series of three-burst pulses presented at 5Hz, 10 pulses/s, 10 pulses/train, 20 trains, 10.0 s intertrain interval; 80% RMT, MagPro) using a figure 8 coil (Coil Cool B65 A/P).

#### Continuous ThetaBurst Stimulation (cTBS) to the r.IFG

The total duration of the cTBS protocol (24) was 34 s. Participants received stimulation over the r.IFG (an intermittent series of three-burst pulses presented at 6 Hz, 18 pulses/s, 600 pulses/train, .1 s intertrain interval; 80% RMT, MagPro) using a figure 8 coil (Coil Cool B65 A/P).

### Statistical Analysis

Each of three task events of interest were modeled separately with a 2 (Time: pre, post) x 2 (Condition: iTBS, cTBS) repeated measures analysis of covariance (rmANCOVA). Though nicotine dependence severity, as measured by the FTND was not significantly associated with inhibitory control performance at any one of the measurement time-points (all p’s > .05), FTND score was entered as a covariate in the rmANOVA to control for variability in this small sample. Significance was defined at α=.05. Given research suggesting cannabis use may account for significant variance in the relationship between nicotine use and inhibitory control (31), and urine drug screen (UDS) results in the current study revealing that 50% (N = 6) of the study sample tested positive for cannabis at the screening visit, baseline task performance differences between UDS outcome groups was assessed. No significant group differences were observed on task performance i.e. trial accuracy (go trials: t=.363; rare trials: t=.020; nogo trials: t =.996; all p’s > .35). Therefore, UDS status was not included as a between subjects variable during hypothesis testing.

## Results

*Blind success.* The double-blind procedure used in the study was successful. Neither the participants [*X* ^2^ (2, N=12) = 1.71, p =.424] nor the researcher administering the behavioral assessments [*X* ^2^ (2, N=12) = 1.54, p =.462] were able to identify TBS conditions.

### Effects of TBS on Inhibitory Control Task Performance

#### Inhibitory Control Trials

**Figure 2:**
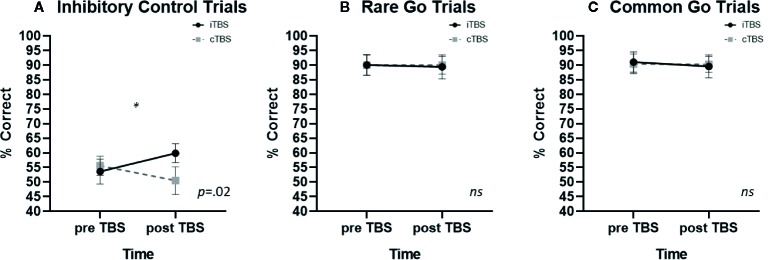
The effects of thetaburst TMS on inhibitory control task performance (mean ± SEM) on **(A)** inhibitory control, **(B)** rare go, and **(C)** common go trials. TMS, transcranial magnetic stimulation.

A significant condition x time interaction was identified on inhibitory control trials [F (1,10) = 7.27, *p* =.022, η^2^ =.421). iTBS improved inhibitory control whereas cTBS impaired inhibitory control (iTBS: pre- *M* = 53.6, SE ± 4.2, post- *M* = 59.88 ± 3.2; cTBS: pre- *M* = 55.6 ± 3.3, post- *M* = 50.5 ± 4.7 **Figure 2A**). No significant main effect of condition or time were observed (p’s > .4). Post-hoc examination of associations between baseline FTND scores and TBS induced change in inhibitory control performance (pre-post TBS Δ) failed to reveal a significant association (iTBS: *p* =.163; cTBS: *p* =.063); omitting FTND in the rmANOVA resulted in a reduction in the effect size (η^2^ =.193).

#### Rare Go Trials

There were no significant effects of TBS on rare go trial performance. Neither the condition x time interaction (*p* =.932; iTBS: pre—*M* = 90.1 ± 3.6, post—*M* = 89.4 ± 4.1; cTBS: pre- *M* = 90.0 ± 3.4, post- *M* = 90.0 ± 3.1; **Figure 2B**) main effect of condition (p=.937) or main effect of time (p=.294) were significant.

#### Frequent Go Trials

There were no significant effects of TBS on frequent go trial performance. Neither the condition x time interaction (p=.750; iTBS: pre—M= 91.1 ± 3.5, post—M = 89.6 ± 3.9; cTBS: pre M = 90.4 ± 3.4, post M = 90.3 ± 2.8; **Figure 2C**), main effect of condition (p=.847) or main effect of time (p=.468) were significant.

## Discussion

Results from the current study confirm that TBS can be used to parametrically modulate inhibitory control among nicotine dependent smokers. Specifically, results from this study revealed that r.IFG iTBS significantly enhances inhibitory control, whereas r.IFG cTBS significantly attenuates inhibitory control; while neither modulated novelty detection or motor responding. The extant reports in the literature for using brain stimulation to modulate executive function have primarily focused on stimulation to the left dorsolateral PFC (32). However, there is overwhelming evidence that the r.IFG (ventrolateral PFC) is a critical cortical node in the network responsible for initiating “stop” signals related to a prepotent motor response (33), and yet there is a dearth of studies reporting on the effects of TMS in general on r.IFG and, to the best our knowledge, these findings are the first to demonstrate that non-invasive neural stimulation using iTBS to the r.IFG enhances baseline inhibitory control among individuals with a substance use disorder.

Inhibitory control—the ability to withhold a prepotent response in favor of performing context-relevant, goal-directed behavior (34)—is proposed to be carried out *via* the hyperdirect pathway composed of glutamatergic mediated excitation from the prefrontal cortex (PFC) to subthalamic nucleus (STN) and then to pallidum, in turn exerting GABAergic mediated inhibition from the pallidum to the thalamus (35). In human neuroimaging studies, evidence of these interconnections has been demonstrated with tractography (36) and functional evidence for the circuitry involvement in IC performance by combined task-based fMRI and electrocorticography (ECoG) (14), IC task-based effective connectivity (37) and lesion studies (38). This neural model is further substantiated by an effective connectivity study revealing that successful IC involves the r.IFG modulating the strength of the excitatory action of the preSMA on STN, which inhibits, *via* the pallidum, motor output from thalamus to motor cortex (37). Taken together, these findings support the existence of an inhibitory control pathway in which the r.IFG serves as a cortical mediated inhibitory command input and the thalamus serves as a final gateway prior to the motor cortex mediated output required to proactively inhibit a prepotent behavioral response.

In the context of tobacco use disorder, smokers exhibit anatomical and functional abnormalities in the r.IFG. Compared to nonsmokers, smokers exhibit less gray-matter volume (GMV) in the IFG (39–41) as well as greater r.IFG BOLD response during neurocognitive task probes of executive function (15, 17). Acute smoking abstinence further increases r.IFG BOLD response during inhibitory control (6) and other neurocognitive tasks (16–18). Abnormalities in IFG structure and function implicate a compensatory mechanism by which smokers may ‘over-recruit’ the r.IFG in an attempt to exert IC. Indeed, baseline inhibitory control task based hyperactivity in the r.IFG is associated with worse smoking cessation outcomes. Moreover, the strength of inhibitory control task-based functional connectivity (tbFC) between the r.IFG and thalamus (corticothalamic circuit) mediates the association between IC task performance and smoking outcomes (9). Based upon the tenet that iTBS induces LTP and strengthens network activity, these findings suggest that the observed effects of r.IFG iTBS on enhancing inhibitory control may have occurred by way of strengthening corticothalamic tbFC. Similarly, the effects of r.IFG cTBS on attenuating inhibitory control may have resulted from a weakening of corticothalamic tbFC. However, further research is needed to directly examine the potential parametric effects of TBS on corticothalamic tbFC.

## Conclusion and Limitations

There is critical need for a principled examination of the neural underpinnings of the effects of TBS on inhibitory control. There is an equally pressing need to evaluate the potential of this technique for modulating inhibitory control circuits implicated in SUD-relevant behavior [however, see Liu et al. (31)]. As demonstrated for the first time here, IC is amenable to enhancement through r.IFG iTBS. The application of r.IFG iTBS to individuals with a SUD, and tobacco use disorder in particular, represents a mechanistically novel path of research, complementing and building upon prior work identifying a systems-level predictive model of relapse vulnerability (9) and with potential utility in improving smoking cessation outcomes. The rationale for selecting the r.IFG as the target region to examine the effects of TBS on IC among smokers is guided by the breadth of literature on the importance of the r.IFG in IC and our prior work with smokers that demonstrated disruption in r.IFG response during tasks probing inhibitory control (9, 15–18). However, we acknowledge that TBS to the pre-SMA—a cortical node in the corticothalamic pathway—has been shown to impact inhibitory control task performance among healthy control participants (26, 42, 43) and therefore also warrants examination in future studies. It is important to note that acute administration of nicotine is known to improve executive function in both smokers and nonsmokers (44), including novelty detection which, in addition to inhibitory control, is also subserved by that r.IFG (45). Given that r.IFG mediates both inhibitory control and novelty detection, the current study utilized a variant of the go/nogo task i.e. Go-Go/NoGo that includes a rare novel item in order to examine whether the effect of TBS to r.IFG is process general or specific to IC. Results from the current study demonstrating that r.IFG TBS parametrically modulates inhibitory control but not novelty detection are fascinating and require further investigation using both fMRI and TBS to better characterize the effects of TBS on process-dependent neurocicuitry function.

The current study sought to compare the effects of iTBS vs. an active condition—cTBS and baseline in order to test the hypothesis that TBS to the rIFG may causally modulate IC in a stimulation-dependent manner (i.e. improve following iTBS, worsen following cTBS). Though a sham condition was considered, we concluded that the sham condition adds little benefit above and beyond the active—cTBS condition (and baseline) for the purpose of testing the study hypothesis. However, future larger-scale clinical studies evaluating iTBS for improving IC and clinical outcomes among individuals with tobacco use disorder will benefit from including a sham condition as a comparator condition. Despite the limitations of the current study, including a relatively small N, brief testing period and demographic and baseline variability among the study sample, the current study results provide novel and promising early-phase evidence that rIFG iTBS may help improve IC among individuals with tobacco use disorder.

## Data Availability Statement

The data that support the findings of this study are available from the corresponding author upon reasonable request.

## Ethics Statement

The studies involving human participants were reviewed and approved by the Medical University of South Carolina IRB. The patients/participants provided their written informed consent to participate in this study.

## Author Contributions

BF designed the study. RN-N, MG, and PM oversaw data collection and data analysis. BF was responsible for data interpretation. RN-N and BF developed the manuscript.

## Funding

This research was supported by NIDA grants R01DA033459, R01DA038700 and UG3DA048510 (BF); in part by pilot research funding, Hollings Cancer Center’s Cancer Center Support Grant P30CA138313 at the Medical University of South Carolina.

## Conflict of Interest

BF is a consultant for Promentis Pharmaceuticals, Inc. for work unrelated to the content of the manuscript.

The remaining authors declare that the research was conducted in the absence of any commercial or financial relationships that could be construed as a potential conflict of interest.

## References

[1] Hester R Lubman DI Yucel M The role of executive control in human drug addiction. Curr Top Behav Neurosci (2010) 3:301–18. 10.1007/7854_2009_28 21161758

[2] Moeller SJ Bederson L Alia-Klein N Goldstein RZ Neuroscience of inhibition for addiction medicine: from prediction of initiation to prediction of relapse. Prog Brain Res (2016) 223:165–88. 10.1016/bs.pbr.2015.07.007 PMC5319822 26806776

[3] Dinur-Klein L Kertzman S Rosenberg O Kotler M Zangen A Dannon PN Response inhibition and sustained and attention in Heavy smokers versus non-smokers. Isr J Psychiatry Relat Sci (2014) 51(4):240–6. 25841219

[4] Luijten M Littel M Franken IH Deficits in inhibitory control in smokers during a Go/NoGo task: an investigation using event-related brain potentials. PloS One (2011) 6(4):e18898. 10.1371/journal.pone.0018898 21526125 PMC3081309

[5] Nestor L McCabe E Jones J Clancy L Garavan H Differences in “bottom-up” and “top-down” neural activity in current and former cigarette smokers: Evidence for neural substrates which may promote nicotine abstinence through increased cognitive control. Neuroimage (2011) 56(4):2258–75. 10.1016/j.neuroimage.2011.03.054 21440645

[6] Kozink RV Kollins SH McClernon FJ Smoking withdrawal modulates right inferior frontal cortex but not presupplementary motor area activation during inhibitory control. Neuropsychopharmacology (2010) 35(13):2600–6. 10.1038/npp.2010.154 PMC2978758 20861830

[7] Powell JH Pickering AD Dawkins L West R Powell JF Cognitive and psychological correlates of smoking abstinence, and predictors of successful cessation. Addict Behav (2004) 29(7):1407–26. 10.1016/j.addbeh.2004.06.006 15345273

[8] Powell J Dawkins L West R Powell J Pickering A Relapse to smoking during unaided cessation: clinical, cognitive and motivational predictors. Psychopharmacol (Berl) (2010) 212(4):537–49. 10.1007/s00213-010-1975-8 20703450

[9] Froeliger B McConnell PA Bell S Sweitzer M Kozink RV Eichberg C Association Between Baseline Corticothalamic-Mediated Inhibitory Control and Smoking Relapse Vulnerability. JAMA Psychiatry (2017) 74(4):379–86. 10.1001/jamapsychiatry.2017.0017 PMC5562280 28249070

[10] Mueller ET Landes RD Kowal BP Yi R Stitzer ML Burnett CA Delay of smoking gratification as a laboratory model of relapse: effects of incentives for not smoking, and relationship with measures of executive function. Behav Pharmacol (2009) 20(5-6):461–73. 10.1097/FBP.0b013e3283305ec7 PMC2886581 19741301

[11] Austin AJ Duka T Rusted J Jackson A Effect of varenicline on aspects of inhibitory control in smokers. Psychopharmacol (Berl) (2014) 231(18):3771–85. 10.1007/s00213-014-3512-7 24652107

[12] Rhodes JD Hawk LW Jr. Ashare RL Schlienz NJ Mahoney MC The effects of varenicline on attention and inhibitory control among treatment-seeking smokers. Psychopharmacol (Berl) (2012) 223(2):131–8. 10.1007/s00213-012-2700-6 22526531

[13] Aron AR Robbins TW Poldrack RA Inhibition and the right inferior frontal cortex: one decade on. Trends Cognit Sci (2014) 18(4):177–85. 10.1016/j.tics.2013.12.003 24440116

[14] Swann NC Cai W Conner CR Pieters TA Claffey MP George JS Roles for the pre-supplementary motor area and the right inferior frontal gyrus in stopping action: electrophysiological responses and functional and structural connectivity. Neuroimage (2012) 59(3):2860–70. 10.1016/j.neuroimage.2011.09.049 PMC3322194 21979383

[15] Froeliger B Modlin LA Kozink RV Wang L Garland EL Addicott MA Frontoparietal attentional network activation differs between smokers and nonsmokers during affective cognition. Psychiatry Res (2013) 211(1):57–63. 10.1016/j.pscychresns.2012.05.002 23154092 PMC3557750

[16] Froeliger B Modlin L Wang L Kozink RV McClernon FJ Nicotine withdrawal modulates frontal brain function during an affective Stroop task. Psychopharmacol (Berl) (2012) 220(4):707–18. 10.1007/s00213-011-2522-y PMC3619410 21989805

[17] Froeliger B Modlin LA Kozink RV Wang L McClernon FJ Smoking abstinence and depressive symptoms modulate the executive control system during emotional information processing. Addict Biol (2012) 17(3):668–79. 10.1111/j.1369-1600.2011.00410.x PMC3288802 22081878

[18] Kozink RV Lutz AM Rose JE Froeliger B McClernon FJ Smoking withdrawal shifts the spatiotemporal dynamics of neurocognition. Addict Biol (2010) 15(4):480–90. 10.1111/j.1369-1600.2010.00252.x PMC2996612 21040240

[19] Hong YH Wu SW Pedapati EV Horn PS Huddleston DA Laue CS Safety and tolerability of theta burst stimulation vs. single and paired pulse transcranial magnetic stimulation: a comparative study of 165 pediatric subjects. Front Hum Neurosci (2015) 9:29. 10.3389/fnhum.2015.00029 25698958 PMC4316715

[20] Oberman L Edwards D Eldaief M Pascual-Leone A Safety of theta burst transcranial magnetic stimulation: a systematic review of the literature. J Clin Neurophysiol (2011) 28(1):67–74. 10.1097/WNP.0b013e318205135f 21221011 PMC3260517

[21] Huang YZ Edwards MJ Rounis E Bhatia KP Rothwell JC Theta burst stimulation of the human motor cortex. Neuron (2005) 45(2):201–6. 10.1016/j.neuron.2004.12.033 15664172

[22] Benali A Trippe J Weiler E Mix A Petrasch-Parwez E Girzalsky W Theta-burst transcranial magnetic stimulation alters cortical inhibition. J Neurosci (2011) 31(4):1193–203. 10.1523/JNEUROSCI.1379-10.2011 PMC6623597 21273404

[23] Klimesch W Doppelmayr M Russegger H Pachinger T Theta band power in the human scalp EEG and the encoding of new information. Neuroreport (1996) 7(7):1235–40. 10.1097/00001756-199605170-00002 8817539

[24] Goldsworthy MR Pitcher JB Ridding MC A comparison of two different continuous theta burst stimulation paradigms applied to the human primary motor cortex. Clin Neurophysiol (2012) 123(11):2256–63. 10.1016/j.clinph.2012.05.001 22633917

[25] Drummond NM Cressman EK Carlsen AN Offline continuous theta burst stimulation over right inferior frontal gyrus and pre-supplementary motor area impairs inhibition during a go/no-go task. Neuropsychologia (2017) 99:360–7. 10.1016/j.neuropsychologia.2017.04.007 28391033

[26] Obeso I Robles N Marron EM Redolar-Ripoll D Dissociating the Role of the pre-SMA in Response Inhibition and Switching: A Combined Online and Offline TMS Approach. Front Hum Neurosci (2013) 7:150. 10.3389/fnhum.2013.00150 23616761 PMC3629293

[27] Heatherton TF Kozlowski LT Frecker RC Fagerstrom KO The Fagerstrom Test for Nicotine Dependence: a revision of the Fagerstrom Tolerance Questionnaire. Br J Addict (1991) 86(9):1119–27. 10.1111/j.1360-0443.1991.tb01879.x 1932883

[28] Chikazoe J Jimura K Asari T Yamashita K Morimoto H Hirose S Functional dissociation in right inferior frontal cortex during performance of go/no-go task. Cereb Cortex (2009) 19(1):146–52. 10.1093/cercor/bhn065 18445602

[29] Friston KJ Holmes A Poline JB Price CJ Frith CD Detecting activations in PET and fMRI: levels of inference and power. Neuroimage (1996) 4(3 Pt 1):223–35. 10.1006/nimg.1996.0074 9345513

[30] Borckardt JJ Nahas Z Koola J George MS Estimating resting motor thresholds in transcranial magnetic stimulation research and practice: a computer simulation evaluation of best methods. J ECT (2006) 22(3):169–75. 10.1097/01.yct.0000235923.52741.72 16957531

[31] Liu Y van den Wildenberg WPM de Graaf Y Ames SL Baldacchino A Is (poly-) substance use associated with impaired inhibitory control? A mega-analysis controlling for confounders. Neurosci Biobehav Rev (2019) 105:288–304. 10.1016/j.neubiorev.2019.07.006 31319124

[32] Dunlop K Hanlon CA Downar J Noninvasive brain stimulation treatments for addiction and major depression. Ann N Y Acad Sci (2017) 1394(1):31–54. 10.1111/nyas.12985 26849183 PMC5434820

[33] Wessel JR Aron AR On the Globality of Motor Suppression: Unexpected Events and Their Influence on Behavior and Cognition. Neuron (2017) 93(2):259–80. 10.1016/j.neuron.2016.12.013 PMC5260803 28103476

[34] Diamond A Executive functions. Annu Rev Psychol (2013) 64:135–68. 10.1146/annurev-psych-113011-143750 PMC4084861 23020641

[35] Jahanshahi M Obeso I Rothwell JC Obeso JA A fronto-striato-subthalamic-pallidal network for goal-directed and habitual inhibition. Nat Rev Neurosci (2015) 16(12):719–32. 10.1038/nrn4038 26530468

[36] Aron AR Behrens TE Smith S Frank MJ Poldrack RA Triangulating a cognitive control network using diffusion-weighted magnetic resonance imaging (MRI) and functional MRI. J Neurosci (2007) 27(14):3743–52. 10.1523/JNEUROSCI.0519-07.2007 PMC6672420 17409238

[37] Rae CL Hughes LE Anderson MC Rowe JB The prefrontal cortex achieves inhibitory control by facilitating subcortical motor pathway connectivity. J Neurosci (2015) 35(2):786–94. 10.1523/JNEUROSCI.3093-13.2015 PMC4293423 25589771

[38] Aron AR Fletcher PC Bullmore ET Sahakian BJ Robbins TW Stop-signal inhibition disrupted by damage to right inferior frontal gyrus in humans. Nat Neurosci (2003) 6(2):115–6. 10.1038/nn1003 12536210

[39] Brody AL Mandelkern MA Jarvik ME Lee GS Smith EC Huang JC Differences between smokers and nonsmokers in regional gray matter volumes and densities. Biol Psychiatry (2004) 55(1):77–84. 10.1016/S0006-3223(03)00610-3 14706428

[40] Fritz HC Wittfeld K Schmidt CO Domin M Grabe HJ Hegenscheid K Current smoking and reduced gray matter volume-a voxel-based morphometry study. Neuropsychopharmacology (2014) 39(11):2594–600. 10.1038/npp.2014.112 PMC4207339 24832823

[41] Gallinat J Meisenzahl E Jacobsen LK Kalus P Bierbrauer J Kienast T Smoking and structural brain deficits: a volumetric MR investigation. Eur J Neurosci (2006) 24(6):1744–50. 10.1111/j.1460-9568.2006.05050.x 17004938

[42] Obeso I Wilkinson L Teo JT Talelli P Rothwell JC Jahanshahi M Theta burst magnetic stimulation over the pre-supplementary motor area improves motor inhibition. Brain Stimul (2017) 10(5):944–51. 10.1016/j.brs.2017.05.008 28624346

[43] Lee HW Lu MS Chen CY Muggleton NG Hsu TY Juan CH Roles of the pre-SMA and rIFG in conditional stopping revealed by transcranial magnetic stimulation. Behav Brain Res (2016) 296:459–67. 10.1016/j.bbr.2015.08.024 26304720

[44] Froeliger B Gilbert DG McClernon FJ Effects of nicotine on novelty detection and memory recognition performance: double-blind, placebo-controlled studies of smokers and nonsmokers. Psychopharmacol (Berl) (2009) 205(4):625–33. 10.1007/s00213-009-1571-y 19488741

[45] Ranganath C Rainer G Neural mechanisms for detecting and remembering novel events. Nat Rev Neurosci (2003) 4(3):193–202. 10.1038/nrn1052 12612632

